# Resistance exercise causes greater serum hepcidin elevation than endurance (cycling) exercise

**DOI:** 10.1371/journal.pone.0228766

**Published:** 2020-02-27

**Authors:** Kazushige Goto, Chihiro Kojima, Nobukazu Kasai, Daichi Sumi, Nanako Hayashi, Hyejung Hwang

**Affiliations:** 1 Graduate School of Sports and Health Science, Ritsumeikan University, Kusatsu, Shiga, Japan; 2 Japan Institute of Sports Sciences, Tokyo, Japan; 3 Physical Activity and Performance Institute, Konkuk University, Seoul, Republic of Korea; National Taipei University of Nursing and Health Sciences, TAIWAN

## Abstract

**Background:**

Hepcidin is an iron regulating hormone, and exercise-induced hepcidin elevation is suggested to increase the risk of iron deficiency among athletes.

**Objective:**

We compared serum hepcidin responses to resistance exercise and endurance (cycling) exercise.

**Methods:**

Ten males [mean ± standard error: 172 ± 2 cm, body weight: 70 ± 2 kg] performed three trials: a resistance exercise trial (RE), an endurance exercise trial (END), and a rest trial (REST). The RE consisted of 60 min of resistance exercise (3−5 sets × 12 repetitions, 8 exercises) at 65% of one repetition maximum, while 60 min of cycling exercise at 65% of $\dot{V}O_{2\max}$ was performed in the END. Blood samples were collected before exercise and during a 6-h post-exercise (0h, 1h, 2h, 3h, 6h after exercise).

**Results:**

Both RE and END significantly increased blood lactate levels, with significantly higher in the RE (*P* < 0.001). Serum iron levels were significantly elevated immediately after exercise (*P* < 0.001), with no significant difference between RE and END. Both the RE and END significantly increased serum growth hormone (GH), cortisol, and myoglobin levels (*P* < 0.01). However, exercise-induced elevations of GH and cortisol were significantly greater in the RE (trial × time: *P* < 0.001). Plasma interleukin-6 (IL-6) levels were significantly elevated after exercise (*P* = 0.003), with no significant difference between the trials. Plasma hepcidin levels were elevated after exercise (*P* < 0.001), with significantly greater in the RE (463 ± 125%) than in the END (137 ± 27%, *P* = 0.03). During the REST, serum hepcidin and plasma IL-6 levels did not change significantly.

**Conclusion:**

Resistance exercise caused a greater exercise-induced elevation in hepcidin than did endurance (cycling) exercise. The present findings indicate that caution will be required to avoid iron deficiency even among athletes in strength (power) types of events who are regularly involved in resistance exercise.

## Introduction

Iron deficiency is a frequently observed diagnosis among athletes [1–4]. Exercise-induced iron deficiency has been attributed to several factors, including sweating, hemolysis, hematuria, and gastrointestinal bleeding [5]. However, over the past decade, an increasing amount of attention has been focused on the influence of hepcidin (a liver-derived iron-regulating hormone) on iron metabolism [6,7]. Hepcidin is a master regulator of iron metabolism [8] that triggers the degradation of ferroportin (an iron export protein) both in the intestine and on the surfaces of macrophages [9,10], thereby reducing dietary iron absorption and iron release from macrophages (impaired iron recycling from damaged erythrocytes). Therefore, increased hepcidin compromises iron availability [11,12]. Many previous studies revealed that exercise acutely increased hepcidin levels, peaking around 3 h after exercise completion [7,13,14]. The exercise-induced hepcidin elevation creates a period of reduced iron absorption, which may compromise an athlete’s iron status [15]. Although hepcidin is augmented by several physiological factors, exercise-induced interleukin-6 (IL-6) is suggested to be an important stimulus for increasing hepcidin production [10]. Furthermore, Peeling et al. [16] recently demonstrated that baseline serum ferritin and iron levels, post-exercise IL-6 levels, and exercise duration explained ~77% of the post-exercise (3 h) increase in hepcidin levels in athletes. According to Dominguez et al. [17], endurance exercise at moderate or vigorous intensity stimulates increases in hepcidin levels between 0h and 6h after completing exercise.

Although exercise-induced hepcidin elevation is well substantiated, the majority of previous studies utilized endurance types of exercise [7,14,18]. To our knowledge, only two previous studies [19,20] have focused on hepcidin response to repeated bouts of maximal cycle sprint exercise (a typical form of anaerobic exercise). Antosiewicz et al. [19] found that three repeated bouts of 30-s maximal cycle sprint exercise significantly increased serum hepcidin levels 1 h after exercise. We recently reported that 15 × 6-s maximal cycle sprint exercise increased hepcidin levels [20]. However, the impact of resistance exercise (another typical form of anaerobic exercise) on hepcidin elevation has not yet been determined in humans. In an animal study [21], 6 weeks of resistance exercise decreased iron absorption, and the authors suggested that elevated hepcidin may be involved in the impaired iron absorption. However, the exercise modality used in the above study was a “climbing exercise”, which is different from general forms of resistance exercise in humans. Considering that exercise-induced hepcidin elevation is associated with energy expenditure during exercise [22], it is unlikely that resistance exercise promotes greater hepcidin production than endurance exercise, due to its lower energy expenditure. In contrast, resistance exercise may elicit hepcidin production mediated by a reduction of muscle glycogen content [23] and subsequent IL-6 production (a stimulating factor of hepcidin elevation) [24]. Because most of athletes, including team sport athletes, endurance athletes, incorporate resistance exercise into their daily training program, determination of resistance exercise-induced hepcidin elevation is valuable.

Therefore, the present study was designed to compare exercise-induced hepcidin responses during a 6-h post-exercise period between resistance exercise and endurance exercise in recreationally trained males. Our first hypothesis was that resistance exercise would initially increase plasma IL-6 levels, with delayed elevation of plasma hepcidin levels. Our second hypothesis was that the magnitude of increase would be similar for resistance exercise and endurance exercise.

## Methods

### Subjects

Ten recreationally trained males [mean ± standard error (SE), age: 23 ± 1 years, height: 172 ± 2 cm, body weight: 70 ± 2 kg] participated in the present study. They were physically active and had several years of experience undergoing regular exercise for both resistance training and endurance training. Each subject was informed of the purpose of the study, experimental procedures, and the possible risks involved in the study, and written informed consent was obtained. The present study was approved by the Ethical Committee for Human Experiments at Ritsumeikan University, in accordance with the Declaration of Helsinki.

### Experimental overview

The subjects visited our laboratory five times throughout the experimental period. On the first visit, the one repetition maximum (1RM) was evaluated for eight exercises. On the second visit, maximal oxygen uptake ($\dot{V}O_{2\max}$) was determined. During the third through fifth visits, three main experiments (including two exercise trials and one rest trial) were conducted. For the main experiments, subjects performed one of three trials at each visit, consisting of a resistance exercise trial (RE), endurance exercise trial (END), or rest trial (REST). The order of the three trials was randomized, with an interval of at least one week between the trials. Strenuous exercise aside from daily physical activity (e.g., walking, commuting) was not allowed during 24h before the main experiments. Furthermore, the subjects were requested to finish standard dinner by 2100 on the day prior to the main experiments.

In the RE and END, blood samples were collected before exercise, immediately after exercise, and 0.5, 1, 2, 3, 4, and 6 h after completing 60 min of exercise to compare the time course of changes in plasma hepcidin, metabolites, and hormonal responses. In the REST, blood samples were collected before the rest, and 3 h after 60 min of rest (identical time point to 3 h after completing exercises in the RE and END) to evaluate diurnal changes in plasma hepcidin and IL-6 levels.

### Evaluations of 1RM and $\dot{V}O_{2\max}$

On the first visit, the 1RM for eight exercises (chest press, lat pull-down, leg press, knee extension, seated row, shoulder press, arm curl, and triceps press-down) were evaluated using weight stack machines (Life Fitness, Tokyo, Japan). Before the start of the 1RM tests, the subjects performed stretching exercises (5 min, three types of stretching for upper limb muscles and lower limb muscles). For the 1RM tests, the load was progressively increased until the participant failed to complete a successful lift. The final load that a subject successfully lifted was defined as the 1RM for each exercise.

On the second visit, $\dot{V}O_{2\max}$ was evaluated using a cycle ergometer (828E, Monark, Uppsala, Sweden). After a 5-min warm-up at 30 W, the subjects started pedaling exercise at 60 W. The load was progressively increased by 30 W every 2 min until exhaustion. The test was terminated when the subjects failed to maintain a pedaling frequency of 60 rpm or reached a $\dot{V}O_{2}$ plateau. Respiratory samples were collected and analyzed using an automatic gas analyzer (AE300S, Minato Medical Science Co., Ltd., Tokyo, Japan) to determine $\dot{V}O_{2}$, carbon dioxide output ($\dot{VC}O_{2}$), minute ventilation ($\dot{V}E$), and the respiratory exchange ratio (RER). The respiratory samples were collected breath-by-breath, and the data were averaged every 30 s.

### Main experiments

The main experiments included two exercise trials (RE and END) and a rest trial (REST). All subjects arrived at the laboratory at the same time of the day following an overnight fast (8:00). In the RE, the subjects conducted 60 min of resistance exercise (eight exercises: chest press, lat pull-down, leg press, knee extension, seated rowing, shoulder press, arm curl, and triceps press-down). Each exercise consisted of 12 repetitions, with four sets for chest press and lat pull-down, and three sets for the remaining six exercises at 65% of 1RM. A 2-min rest period was allowed between sets and between exercises.

In the END, the subjects conducted 60-min of pedaling exercise (828E, Monark) at 65% of $\dot{V}O_{2\max}$. Pedaling frequency was set at 60 rpm. All exercises in RE and END were supervised by experimental staffs who understood a common instruction for the prescribed exercises. Furthermore, each exercise and post-exercise measurements in RE and END were conducted at the same time of the day. In the REST, subjects maintained rest at the same laboratory while reading books and watching DVDs. Due to the different exercise modes between RE and END, we were not able to match the energy expenditure between the trials. Alternatively, the exercise duration and the relative exercise intensity for the maximal work capacity (1RM for the RE, $\dot{V}O_{2\max}$ for the END) were matched.

In the RE and END, a prescribed meal (889 kcal, protein 11%, fat 29%, carbohydrate 60%) was provided 2 h (after completing required measurements) after exercise completion. The same meal was provided at the identical time point in the REST.

### Blood sampling and analysis

**Fig 1:**
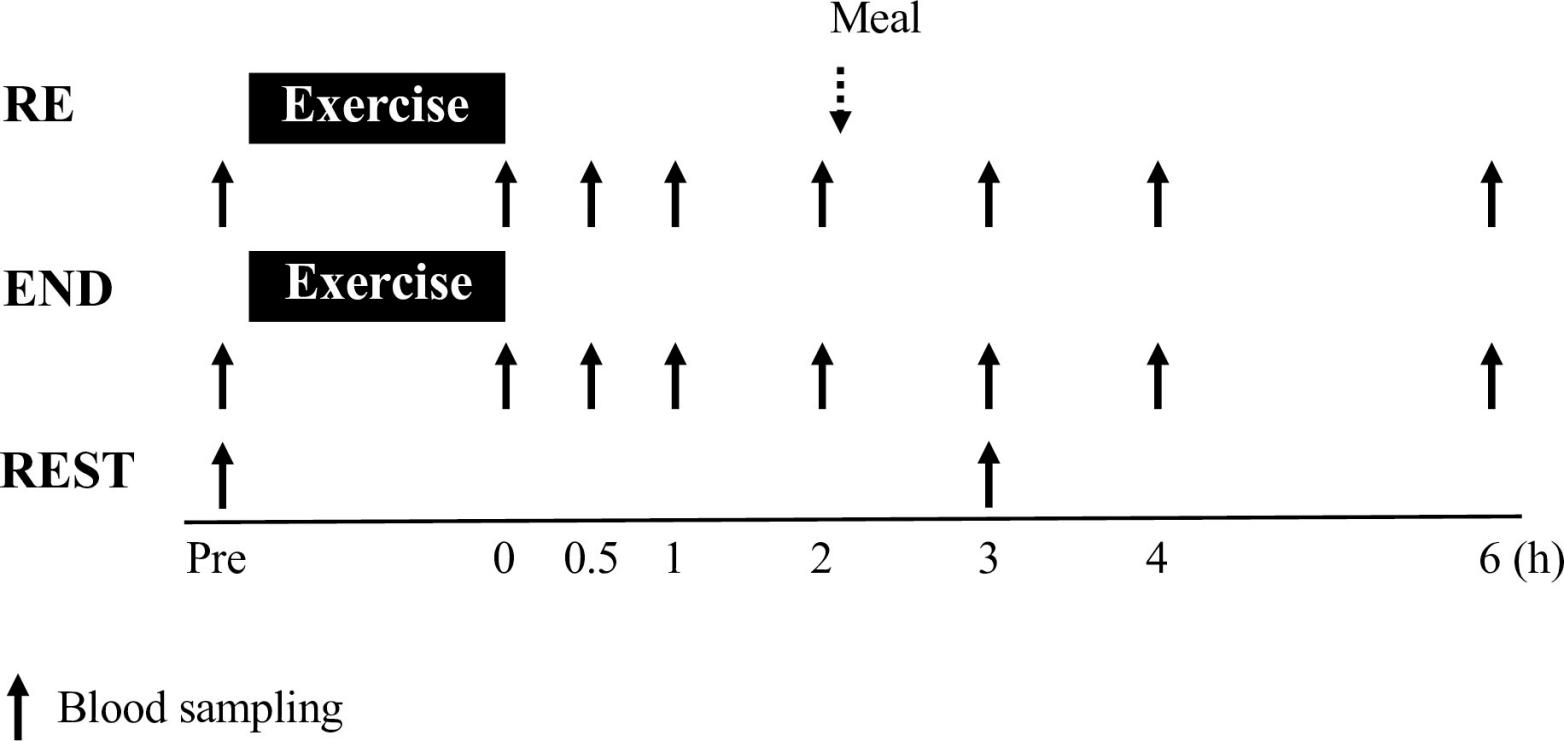
Experimental schedule, indicating the timing of measurements.

In the RE and END, blood samples were collected from an antecubital vein eight times: before exercise, immediately after exercise, 0.5, 1, 2, 3, 4, and 6 h after exercise. In the REST, blood samples were collected at two time points (identical time points to the before exercise and 3 h after exercise time points in the RE and END) (Fig 1). Blood samples were immediately transferred to either the 9 ml of tube for serum separation or 7 ml of tube containing ethylenediaminetetraacetate (EDTA-Na) for plasma separation. After transferring to the tubes, serum samples were put for 20 min under the room temperature to clot. The serum and plasma were obtained after 10 min of centrifugation (3,000 rpm, 4°C) and stored at -80°C until further analysis.

From the obtained blood samples, blood glucose, lactate, plasma hepcidin, and IL-6, and serum growth hormone (GH), cortisol, myoglobin (Mb), and iron levels were evaluated. Serum GH, cortisol (electro chemiluminescence immunoassay method), Mb (chemiluminescent immunoassay), and iron levels (nitroso-PSAP method) were measured at a clinical laboratory (SRL, Tokyo, Japan). The intra-assay coefficients of variation (CV) and the limit of detection for these assays were 2.8% (CV) and 0.03 ng/mL (limit of detection) for serum GH, 5.2% (CV) and 0.06 μg/dL (limit of detection) for cortisol, 6.0% (CV) and 1.0 ng/mL (limit of detection) for serum Mb, and 0.1% (CV) and 6 μg/mL (limit of detection) for serum iron. Plasma hepcidin levels were analyzed with enzyme linked immunosorbent assay (ELISA) using a commercially available kit (R&D Systems, Minneapolis, MN, USA) [25]. The intra-assay CV for the hepcidin assay was 4.2%. Plasma IL-6 levels were also determined with ELISA using a kit (R&D Systems). The intra-assay CV for the IL-6 assay was 3.9%. Blood glucose and lactate levels were measured immediately after blood collection using a glucose analyzer (Freestyle, Nipro Corporation, Osaka, Japan) and a lactate analyzer (Lactate Pro, Arkray, Inc., Kyoto, Japan), respectively.

### Statistical analysis

All data are presented as means ± SE. The main purpose of the present study was to compare the time course of changes in each variable between RE and END. Additionally, due to different measurement points between the two exercise trials (RE and END) and REST, the exercise trials were compared separately to the REST trial. For the comparisons between the RE and END, two-way repeated-measures analysis of variance (ANOVA) was used to confirm the interaction (trial × time) and main effects. When a significant interaction or main effect was evident, a post-hoc Tukey-Kramer test was performed to identify differences. Effect sizes (ES) were calculated to reveal the magnitudes of the differences [partial η^2^ values for repeated-measures two-way ANOVA; Cohen’s d (d) for the paired t-test]. In the REST, a paired *t*-test was applied to confirm differences between the baseline value (Pre) and 3 h after 60 min of rest (corresponding to 3 h after exercise completion in the RE and END). For serum hepcidin and plasma IL-6 levels, exercise-induced relative changes were calculated [relative change (%) = ((the level at respective time point–the level before exercise)/ the level before exercise) ×100) +100]. Furthermore, the area under the curve during 6 h of post-exercise was calculated by the area of trapezoids.

For all tests, a *P*-value < 0.05 was considered to indicate statistical significance.

## Results

### Plasma hepcidin

**Fig 2:**
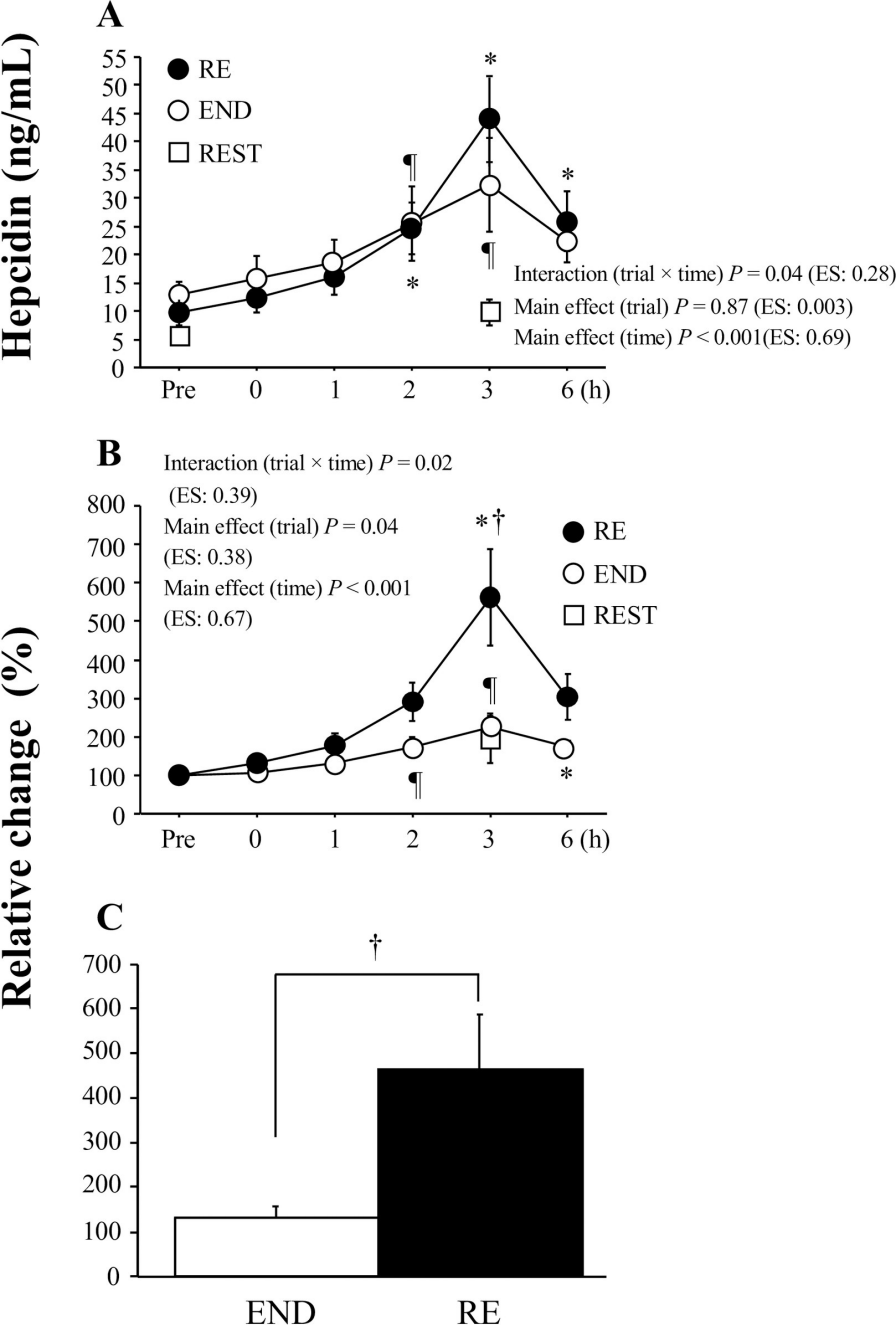
Plasma hepcidin levels before exercise and during the post-exercise period. Absolute levels (A) and relative values compared with the pre-exercise time point (B) are presented. (C) indicates relative change between pre-exercise levels and maximal levels during the post-exercise period in the RE and END. In the REST, plasma hepcidin levels were analyzed only before the start of the rest period and at a time point corresponding to 3 h after exercise in the RE and END. Values present means ± SE. *P < 0.05 vs. Pre (RE). ¶ *P* < 0.05 vs. Pre (END). †*P* < 0.05 between the RE and END.

Fig 2 presents plasma hepcidin levels before exercise and during the 6-h post-exercise period. Both RE and END showed significant increases in plasma hepcidin levels 2 h after exercise (*P* < 0.001 for main effect of time). The RE showed a greater elevation in plasma hepcidin levels, and a significant interaction [trial (RE, END) × time] was observed (*P* = 0.04, Fig 2A). As shown in Fig 2B, a significant interaction [trial (RE, END) × time] was also observed for the relative change from the pre-exercise level (*P* = 0.02). The RE trial induced a significantly higher level 3 h after exercise than the END trial (*P* = 0.03, Fig 2B). The relative change between the pre-exercise level and the peak level during the 6-h post-exercise period was significantly higher in the RE trial (463 ± 125%) than in the END (131 ± 27%, *P* = 0.03, ES = 1.2, Fig 2C).

### Plasma IL-6

**Fig 3:**
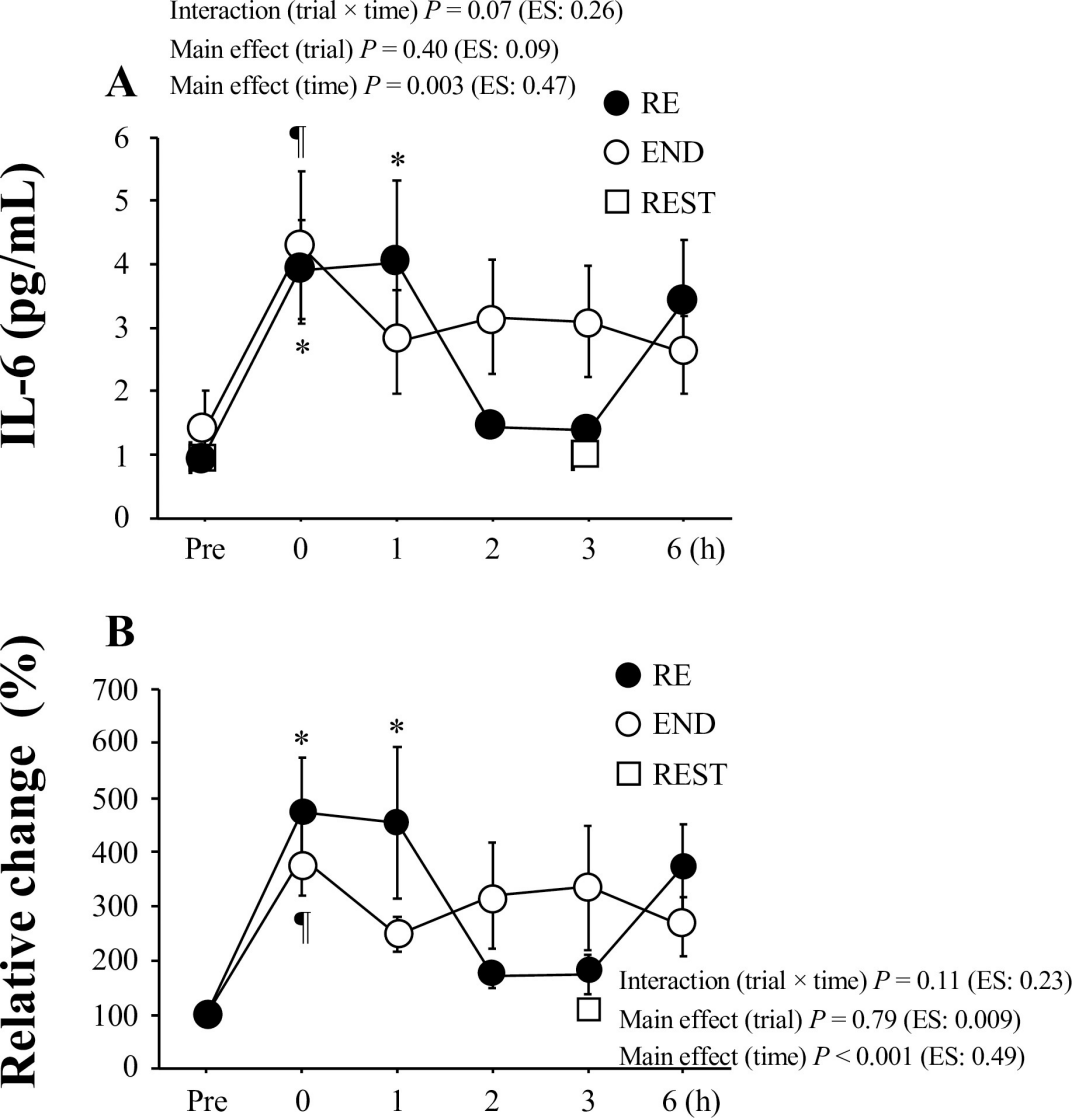
Plasma IL-6 levels before exercise and during the post-exercise period. Absolute levels (A) and relative values compared with the pre-exercise time point (B) are presented. Values present means ± SE. *P < 0.05 vs. Pre (RE). ¶ *P* < 0.05 vs. Pre (END).

Fig 3 presents plasma IL-6 levels before exercise and during the 6-h post-exercise period. Both RE and END showed significant increases in plasma IL-6 levels after exercise (*P* = 0.003 for main effect of time). However, no significant interaction [trial (RE, END) × time] or main effect for trial was observed (Fig 3A). Similarly, there was no significant interaction [trial (RE, END) × time] or main effect for trial (*P* = 0.11 for interaction, *P* = 0.79 for trial) for relative change from the pre-exercise level (Fig 3B). Furthermore, the area under the curve (AUC) of plasma IL-6 did not correlate significantly with the AUC of serum hepcidin in either the RE (r = 0.185, *P* = 0.65) or END (r = 0.54, *P* = 0.14).

### Other blood variables

Table 1 presents blood glucose, lactate, and serum iron levels. For blood glucose levels, a significant interaction [trial (RE, END) × time], *P* = 0.002] and main effects for time (*P* < 0001) and trial (*P* = 0.02) were observed. Both RE and END showed significant increases in blood glucose levels, and the RE trial induced a significantly higher level immediately after exercise (*P* = 0.0007) and 6 h after exercise (*P* = 0.02). For blood lactate levels, a significant interaction [trial (RE, END) × time] and main effects for time and trial were observed (*P* < 0.001). The RE trial caused marked increases in blood lactate levels, and these levels remained significantly higher than those in the END throughout the 6-h post-exercise period (*P* = 0.003). The serum iron levels showed a significant interaction [trial (RE, END) × time, *P* = 0.03]. Serum Fe levels were significantly elevated after exercise in both trials (main effect for time, *P* < 0.001). At 6 h after excise, the END trial showed a tendency toward higher levels (106 ± 9 μg/dL) than the RE trial (83 ± 10 μg/dL, *P* = 0.07).

**Table 1:** Blood glucose, lactate and serum iron levels.

		Pre	0 h	0.5 h	1 h	2 h	3 h	4 h	6 h
Glucose (mmol/L)	RE	4.7 ± 0.1	5.4 ± 0.3†	4.8 ± 03	4.6 ± 0.1	4.9 ± 0.1	6.5 ± 0.3*	63 ± 02*	4.9+ 0.1†
	END	4.5 ± 0.1	4.1 ± 0.1	4.7 ± 0.1	4.7 ± 0.1	4.9 ± 0.2	6.5 ± 0.4¶	5.7 ± 0.2¶	4.6 ± 0.1
Lactate (mmol/L)	RE	1.0 ± 0.2	12.4 ± 0.5*†	6.5 ± 0.5*†	3.1 ± 0.2*†	1.5 ± 0.2†	1.9 ± 0.1†	1.5 ± 0.1†	1.3 ± 0.1†
	END	1.6 ± 03	3.9 ± 0.6*	1.6 ± 0.2	1.1 ± 0.1	1.1 ± 0.2	1.4 ± 0.2	1.1 ± 0.1	0.9 ± 0.1
Iron (lig/mL)	RE	109 ± 14	157 ± 23*	128 ± 15	116 ± 15	115 ± 15	114 ± 14	101 ± 15	83 ± 10
	END	105 ± 8	133 ± 11¶	121 ± 8	119 ± 10	122 ± 10	122 ± 8	120 ± 10	106 ± 9

Value are means ± SE.

* *P* < 0.05 vs. Pre (RE).

¶ *P* < 0.05 vs. Pre (END).

† *P* < 0.05 between RE and END.

**Fig 4:**
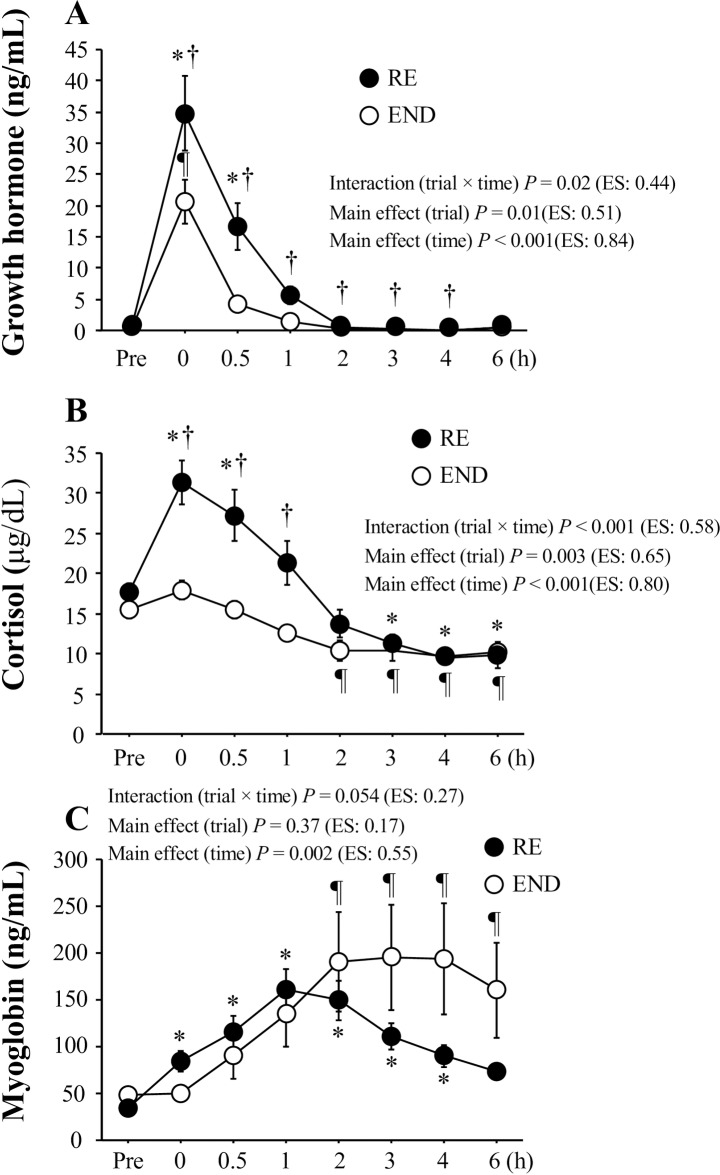
Serum GH, cortisol, and myoglobin levels before exercise and during the post-exercise period. Values present means ± SE. *P < 0.05 vs. Pre (RE). ¶ *P* < 0.05 vs. Pre (END). †*P* < 0.05 between the RE and END.

Fig 4 presents serum GH, cortisol, and Mb levels. Both RE and END induced marked increases in serum GH levels after exercise (main effect for time, *P* < 0.001, ES = 0.84), but the exercise-induced elevation was particularly profound in the RE [trial (RE, END) × time, *P* = 0.02, ES = 0.44; main effect for trial, *P* = 0.02, ES = 0.51, Fig 4A]. For the serum cortisol response, a significant interaction [trial (RE, END) × time, *P* < 0.001, ES = 0.58] and main effects for trial (*P* = 0.003, ES = 0.65) and time (*P* < 0.001, ES = 0.80) were observed. The RE induced significantly higher levels immediately after exercise, and 0.5 (*P* = 0.003) and 1 h after exercise than the END (*P* = 0.003, Fig 4B). Both RE and END showed significant increases in serum Mb levels after exercise (main effect for time, *P* = 0.002, ES = 0.55). However, no significant interaction [trial (RE, END) × time, *P* = 0.054, ES = 0.27] or main effect for trial (*P* = 0.37, ES = 0.17) was observed (Fig 4C).

## Discussion

The main finding of the present study was that 60 min of resistance exercise significantly increased plasma hepcidin levels. Plasma hepcidin levels were maximized 3 h after completing either resistance exercise or endurance exercise (cycling). However, the exercise-induced elevations in hepcidin levels were more profound after resistance exercise than after 60 min of cycling (endurance exercise). To our knowledge, this is the first report indicating that hepcidin is upregulated by a single bout of resistance exercise in humans.

Both RE and END showed significant increases in plasma hepcidin levels 2 h after exercise. In the RE, hepcidin levels remained significantly elevated for 6 h after exercise. Delayed elevation of hepcidin levels after exercise has been frequently reported in previous studies [26,27], although most of these studies utilized running (weight-bearing exercise)-based exercise sessions. In a limited study using cycling exercise (non-weight-bearing exercise) [28], exercise-induced hepcidin elevation did not differ significantly between the running exercise (40 min at 65% of peak running velocity) and cycling exercise (40 min at 65% of peak power output), suggesting that any form of structured endurance exercise (e.g., running or cycling) elevates hepcidin levels, potentially compromising iron metabolism during the post-exercise period. However, in the present study, it is important to note that the RE (463 ± 125% vs. pre-exercise) showed significantly greater elevations of hepcidin levels than the END (131 ± 27% vs. pre-exercise). The effect of anaerobic exercise (e.g., resistance exercise, sprint exercise) on post-exercise hepcidin regulation has not been fully elucidated. In a previous study [19], three repeated bouts of 30-s maximal cycle sprint exercise significantly increased hepcidin levels 1 h after exercise. However, the hepcidin levels in this study were evaluated only 1 h, 24 h, and 5 days after exercise. In our previous study [20], we collected blood samples immediately after exercise and 1 and 3 h after exercise. We found that 15 × 6-s maximal cycle sprint exercise significantly increased serum hepcidin levels 3 h after exercise. However, in the above studies, detailed time-course changes in post-exercise hepcidin responses were not illustrated due to the limited number of post-exercise blood samplings. In the present study, we successfully monitored gradual elevations in hepcidin levels throughout a 6-h post-exercise period, which peaked 3 h after exercise. Consequently, it is likely that different forms of exercise (resistance exercise vs. endurance exercise) do not affect the timing of maximum hepcidin levels during the post-exercise period.

Several physiological factors are considered to explain the augmented hepcidin response observed in the RE. First, the primary mediator for the upregulation of hepcidin secretion is believed to be IL-6 elevation [10]. However, this idea was not supported, because no significant difference in plasma IL-6 levels between the RE and END was observed. Additionally, no significant correlation was observed between the relative changes in in plasma IL-6 level and serum hepcidin level during post-exercise. Second, although both energy expenditure and energy deficit (negative energy balance) resulting from exercise were inversely correlated with hepcidin elevation [22], the energy expenditure appeared to be independent of augmented hepcidin responses in the RE, because energy expenditure was thought to be lower during the RE. Third, baseline iron levels affect exercise-induced hepcidin elevation [16]. Exercise-induced hepcidin levels were impaired in subjects with iron deficiency [29]. We did not evaluate ferritin levels (an indication of iron stores). However, average levels of serum iron (>100 μg/dL) were within normal range, and no subjects under iron deficient status or with anemia were included. Alternatively, post-exercise elevation of serum iron levels due to hemolysis [30] can be a strong stimulus for increasing hepcidin [16]. Notably, post-exercise serum iron levels (an indirect marker of exercise-induced hemolysis) were about 10% higher in the RE than in the END. Therefore, augmented post-exercise iron levels in the RE may explain subsequent elevation of serum hepcidin. Finally, GH stimulates erythropoiesis [31,32], and GH administration suppressed hepcidin levels in a dose-dependent manner [33]. In the present study, post-exercise GH elevation was significantly greater in the RE. Therefore, the inhibitory action of elevated GH levels on hepcidin production was insufficient to attenuate exercise-induced hepcidin elevation. Exercise-induced cortisol elevation was more profound in the RE than in the END, suggesting that exercise-induced catabolic action was augmented following the RE. However, the exercise-induced cortisol response did not correlate with the hepcidin response.

The present study includes some limitations in the interpretation of the results. First, we selected 60 min of cycling exercise for the END. As aforementioned, exercise-induced hemolysis is greater during running (weight-bearing exercise) than during cycling (non-weight-bearing exercise). Therefore, post-exercise hepcidin elevation may differ when running exercise is applied. Several studies [28, 30] have compared hepcidin response between running and cycling, but the findings were not consistent. Second, the cumulative effects of several exercise sessions over consecutive days of training should be explored in the future, as recent studies using 3 days [34] or 7 days of running training [35] or 7 days of military winter training [36] revealed significantly elevated hepcidin levels at rest. Third, we were not able to match energy expenditure during exercise between the RE and END. It was difficult to set the same energy expenditure between the RE and END due to different exercise mode. Although we selected the same exercise duration (60 min) and relative exercise intensity (65%) for the 1RM (RE) and $\dot{V}O_{2\max}$, the difference in energy expenditure might affect the present results. Fourth, in the REST, the blood drawing was limited twice (i.e., Pre, a time point corresponding to 3 h after exercise in the RE and END), since we focused on comparison between the two exercise trials (RE vs. END). In spite of smaller numbers of blood drawing, the result in the REST presented that exercise itself increased serum hepcidin levels. Finally, since we recruited active males as subjects to mimic the situation on sport fields, the outcomes may be different among clinical populations. However, the present findings also suggest that caution will be required to avoid iron deficiency even among athletes in strength (power) types of events who are regularly involved in resistance exercise.

## Conclusion

A 60 min of resistance exercise, consisting of eight exercises for upper and lower body muscles, promoted exercise-induced elevation in hepcidin than a 60 min of endurance (cycling) exercise.
